# Low prevalence of *H. pylori *Infection in HIV-Positive Patients in the Northeast of Brazil

**DOI:** 10.1186/1471-230X-11-13

**Published:** 2011-02-19

**Authors:** Andréa BC Fialho, Manuel B Braga-Neto, Eder JC Guerra, André MN Fialho, Karine C Fernandes, Juliana LM Sun, Christianne FV Takeda, Cícero IS Silva, Dulciene MM Queiroz, Lucia LBC Braga

**Affiliations:** 1 Clinical Research Unity - Department of Internal Medicine - Federal University of Ceará, Fortaleza, Ceará, Brazil; 2 Laboratory of Bacteriology Research - Federal University of Minas Gerais, Belo Horizonte, Minas Gerais, Brazil; 3 São José Hospital, Fortaleza, Ceará, Brazil

## Abstract

**Background:**

This study conducted in Northeastern Brazil, evaluated the prevalence of *H. pylori *infection and the presence of gastritis in HIV-infected patients.

**Methods:**

There were included 113 HIV-positive and 141 age-matched HIV-negative patients, who underwent upper gastrointestinal endoscopy for dyspeptic symptoms. *H. pylori *status was evaluated by urease test and histology.

**Results:**

The prevalence of *H. pylori *infection was significantly lower (p < 0.001) in HIV-infected (37.2%) than in uninfected (75.2%) patients. There were no significant differences between *H. pylori *status and gender, age, HIV viral load, antiretroviral therapy and the use of antibiotics. A lower prevalence of *H. pylori *was observed among patients with T CD4 cell count below 200/mm^3^; however, it was not significant. Chronic active antral gastritis was observed in 87.6% of the HIV-infected patients and in 780.4% of the control group (p = 0.11). *H. pylori *infection was significantly associated with chronic active gastritis in the antrum in both groups, but it was not associated with corpus chronic active gastritis in the HIV-infected patients.

**Conclusion:**

We demonstrated that the prevalence of *H. pylori *was significantly lower in HIV-positive patients compared with HIV-negative ones. However, corpus gastritis was frequently observed in the HIV-positive patients, pointing to different mechanisms than *H. pylori *infection in the genesis of the lesion.

## Background

*Helicobacter pylori *infection is the major etiologic factor of chronic gastritis and peptic ulcer in the general population. Gastrointestinal (GI) symptoms are frequent among patients infected with human immunodeficiency virus (HIV) and with acquired immunodeficiency syndrome (AIDS) [1,2] However, the role of *H. pylori *infection in the GI tract mucosa of HIV patients is not well defined [3]. Some studies suggested that interactions between the immune/inflammatory response, gastric physiology and host repair mechanisms play an important role in dictating the disease outcome in response to *H. pylori *infection, suggesting that the host's immune competence might be an important issue in *H. pylori *infection [4,5].

Data in regard to the prevalence of *H. pylori *infection in HIV-infected population are controversial. Some reports have shown that the rate of the infection in HIV-positive patients is remarkably low when compared with the general population [6,7]. Conversely, other studies have not found similar results [8-10].

It is well known that the immune deficiencies caused by HIV give rise to many different gastrointestinal opportunistic infections, such as cytomegalovirus (CMV) infection and fungal esophagitis [11,12]. However, there are few studies evaluating the gastric mucosa of patients co-infected by *H. pylori *and HIV [13-15].

Therefore, the aim of this study was to evaluate the prevalence of *H. pylori *infection, risk factors associated with the infection, as well as the macroscopic and microscopic alterations of the gastric mucosa of HIV-infected patients in a high *H. pylori *prevalence area in Northeastern, Brazil.

## Methods

The study was approved by the Ethical Committee of Research of the University of Ceará, and informed consent was obtained from each patient. This prospective cross-sectional study was carried out at the Hospital São José, a major referral center for assistance of HIV-infected individuals in the city of Fortaleza, Ceará, Brazil. From May 2001 to April 2003, 113 HIV-positive patients who underwent upper gastrointestinal endoscopy for dyspeptic symptoms were included in the study. The control group was composed by 141 HIV-negative patients who were undergoing upper gastrointestinal endoscopy for investigation of dyspeptic symptoms at the University Hospital Walter Cantideo, Fortaleza, Ceara, Brazil. Patients and age matched controls (interval of 10 years) were enrolled at the same period. All patients gave written informed consent to participate in the study and answered a questionnaire about symptoms and consumption of medications, including acid secretion inhibitors and antibiotics six months before endoscopy. In the HIV-positive patient group, data regarding the risk factors for HIV infection and antiretroviral therapy were also obtained. Total T CD4 cell count and HIV viral load were accepted as valid if the blood sample for their determination had been taken within 1 month before or after the entrance in the study.

### Upper gastrointestinal endoscopy

Gastro-endoscopy was performed with Olympus video endoscopes (Olympus Optical Co, Ltd. GIF TYPE V) in the standard manner. Fragments of the gastric mucosa were obtained from the five sites recommended by the Houston-updated Sydney system for classification of gastritis and to evaluate the presence of spiral microorganism stained by Giemsa [16]. Two fragments from the lesser curvature of the gastric antrum and two from the lesser curvature of the lower gastric body were obtained for urease test. The activity of chronic gastritis was classified as mild, moderate and marked based on the number of neutrophil infiltration. The specimens were fixed in 10% formalin, embedded in paraffin wax, and 5-mm sections were stained with hematoxylin and eosin for histology and with Giemsa staining to evaluate *H. pylori *status.

Exclusion criteria included age below 18 years old or above 80 years old, other serious medical problems, or previous treatment for *H. pylori *infection. *H. pylori *status was determined by the rapid urease test and histology (Giemsa staining) and was considered negative when both tests were negative.

### Statistical Analysis

Data were analyzed using the software SPSS (version 10.0, Chicago, IL). Chi square test with Yates' correction or Fischer's exact test were used to compare results among the different groups. Significance was accepted at P values below 0.05.

## Results

Two hundred and fifty four subjects were included: 113 HIV-infected patients and 141 age-matched controls. The mean age of HIV infected patients was 36.0 years (range, 21-70 years) and 61.9% (70/113) were male. The mean age of the control group was 39.7 years (range 18-76 years) and 36.2% (51/141) were male. Most of the symptoms of HIV-positive patients were nonspecific, such as diarrhea, dyspepsia, abdominal pain, nausea, vomiting, odynophagia or dysphagia. The frequency of diarrhea, odynophagia, and dysphagia was significantly higher in HIV-positive group compared with the controls (*P *< 0.05).

Macroscopic lesions in the HIV-infected group included, widespread esophageal candidiasis (32.7%; 37/113), esophageal ulcers (7.9%; 9/113) and candidiasis plus esophageal ulcers (1.7%; 2/113). *Cryptosporidium *was found in the gastric mucosa of two HIV-infected patients. Table 1 shows the endoscopic gastric mucosal findings in HIV-positive and HIV-negative dyspeptic patients. Corpus gastritis was significantly more frequently observed in the dyspeptic HIV-positive than in HIV-negative patients.

**Table 1:** Endoscopic findings of the gastroduodenal mucosa of dyspeptic HIV-positive and negative patients

Endoscopic findings	HIV + (n = 113	HIV - (n = 141)	Total	*p*-value
	N (%)	N (%)	N	
Normal	0 (18.0)	30 (22.0)	50	0.57
Antral gastritis	44 (38.9)	41 (29.1)	85	0.10
Corpus Gastritis	42 (37.2)	25 (12.1)	67	<0.001
Pangastritis	3 (2.7)	0	3	0.05
Gastric erosion	16 (14.2)	27 (19.2)	43	0.29
Gastric ulcer	2 (1.7)	3 (2.1)	5	0.83
Duodenitis	5 (4.4)	2 (1.4)	7	0.15
Duodenal erosion	3 (2.7)	2 (1.4)	5	0.84

**Figure 1:**
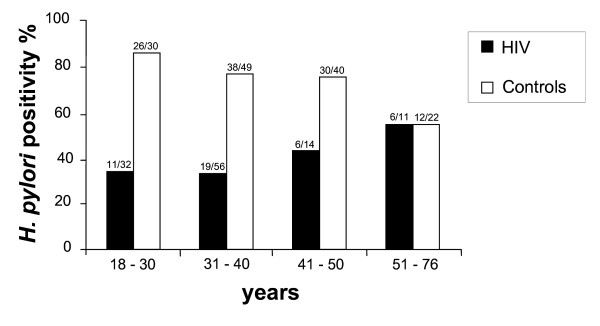
***Helicobacter pylori *infection in HIV-positive and -negative patients according to the age**.

The overall prevalence of *H. pylori *infection was significantly lower (p < 0.001) in HIV-infected patients (37.2%; 42/113) when compared with the controls (75.2%; 106/141); and did not increase with age (p = 0.73). Of note, the infection prevalence in the oldest group did not differ between HIV-positive and HIV-negative patients. The prevalence of *H. pylori *infection in the HIV-positive patients and controls according to the age is shown in Figure 1.

In the HIV-positive group, there was no significant difference between *H. pylori *status and gender, age, HIV viral load, antiretroviral therapy and the use of antibiotics and H2-blocker. Only 4 patients referred the use of proton pump inhibitors (PPI). A non-significant lower prevalence of *H. pylori *infection was observed in the patients with T CD4 cell count below 200 (Table 2).

**Table 2:** Covariates associated with *H. pylori *infection in HIV-positive patients

Variables	*H. pylori *+ N (%)	Total	*p*-value
Gender			
Female	17 (39.5)	43	0.68
Male	25 (35.7)	70	
CD4 count			
≤200	17 (28.8)	59	0.06
>200	25 (46.3)	54	
Viral load			
<100.10^3^	30 (42.8)	71	0.11
≥100. 10^3^	12 (27.9)	42	
Antibiotics			
No	23 (44.2)	52	0.15
Yes	19 (31.1)	61	
Use of H2-blocker			
No	31 (38.3)	76	0.25
Yes	11 (16.6)	37	
Antifungic therapy			
No	36 (40.5)	87	0.09
Yes	6 (29.7)	26	
Antiretroviral therapy HAART			
No	12 (38.3)	42	0.15
Yes	30 (16.6)	71	

The gastric mucosa histological results are shown in Table 3. Chronic active antral gastritis was observed in 87.6% (99/113) of the HIV-infected patients and in 80.1% (113/141; p = 0.11) of the control group. *H. pylori *infection was significantly associated with the presence of chronic active antral gastritis in both groups (p = 0.03 and p < 0.001, respectively). No significant difference (p = 0.89) was also observed between the groups in respect to the frequency of chronic active corpus gastritis (53.1% in the HIV-positive patients and 53.9% in the HIV-negative patient). However, the *H. pylori *infection did not associate with chronic active corpus gastritis in the HIV-positive patients (p = 0.15), but high association was observed in the HIV-negative ones (p < 0.001). Additionally, in the HIV-negative group, the degree of gastritis was also associated with *H. pylori *infection, being the presence of the microorganism more frequently observed in the more marked (50%, 40/80) than in moderated (10%, 2/20) gastritis. Atrophy/intestinal metaplasia was observed less frequently in the gastric corpus of HIV-positive (6.2%, 7/113) than in the gastric corpus of HIV-negative (9.9%, 14/141) patients, but the differences were not significant (p = 0.27).

**Table 3:** Association between the frequency of *H. pylori *infection and antral and corpus active gastritis in HIV-infected patients and controls

	HIV-positive (n = 113)		HIV-negative (n = 141)		*p*-value
	HP+ (n = 42)	HP- (n = 71)	HP+ (n = 106)	HP- (n = 35)	
Chronic active antral gastritis	41 (97.6%)	58 (81.7%)	105 (99.1%)	8 (22.9%)	0.15
	p = 0.03		p < 0.001		
Chronic active corpus gastritis	26 (61.9%)	34 (47.9%)	69 (65.1%)	7 (20.0%)	0.89
	p = 0.15		p < 0.001		

## Discussion

The prevalence of *H. pylori *infection was lower in the HIV-positive group than in the age-matched controls. The low prevalence of *H. pylori *infection we observed in the HIV-positive patients differs profoundly from that previously reported (82.0%) in HIV-negative adults from a poor urban Community in the same city (Fortaleza; Brazil) [17]. A similar result has been observed in a cross-sectional study in Southeastern Brazil that evaluated the prevalence of *H. pylori *infection in 528 HIV-infected patients (32.38% of *H. pylori *positivity) [18]. Studies from East countries, where *H. pylori *infection is highly prevalent such as Taiwan [19] and China [20] also demonstrated a lower *H. pylori *infection prevalence (17.3% and 22.1%, respectively) in HIV-infected than in-non-infected (63.5% and 44.8%, respectively) patients. Conversely, studies from Argentina and from India showed similar *H. pylori *infection prevalence in HIV-infected and non infected patients [13,21,22].

It has to be emphasized that *H. pylori *infection was diagnosed by histology and urease test in all patients. The results were concordant with those obtained by the evaluation of *H. pylori *specific *ure*A gene in the paraffin imbedded gastric tissue from a subgroup of patients (data not shown) and both HIV-positive and -negative patients belong to the same low-income population. As above mentioned, the prevalence of *H. pylori *infection in a similar population from Fortaleza in respect to the socio-economical level is high [17]. It is well known that *H. pylori *infection is mainly acquired during childhood and that once acquired it is life-long lasting [1]. Therefore, we may hypothesize that the HIV-infected patients we studied had been exposed to the bacterium early in life and most of them became infected, but loose the infection after acquired HIV infection. Alternatively, the *H. pylori *gastric load might be decreased in the HIV-positive patients leading to *H. pylori *infection misdiagnosis. Explanations include decreased gastric acid secretion predisposing to gastric colonization by other microorganisms that might compete with *H. pylori*, the use of either antibiotics or PPI and, as suggested in other studies, the low count of T CD4 cells in AIDS patients [6,21,23,24].

It has been suggested that T CD4 cells play a role in inducing or perpetuating tissue and epithelial damage that may facilitate *H. pylori *colonization [25]. In this study, HIV-positive patients were stratified according to the T CD4 cell counts above or below 200 cells/mm3 and a tendency of lower prevalence of *H. pylori *infection was observed in the group of patients with T CD4 cell count of 200 or below.

Hypochlorhydria has been described in HIV-positive patients [23]. Previous studies have shown that HIV-positive patients with overt AIDS have significantly increased serum levels of gastrin and pepsinogen II compared with HIV-positive patients without overt AIDS [26]. Hypochlorhydria may provide a less suitable environment for *H. pylori *and predispose to overgrowth of other bacteria [27]. Inhibition of *H. pylori *by competition with other opportunistic pathogens such as Cytomegalovirus via unknown mechanisms has been also suggested [23,28]. The intragastric environment may be also modified by previous use of PPI. In this study; however, only four HIV-positive patients were under PPI therapy. The frequent usage of antibiotics for treatment or prophylaxis against opportunistic infections in patients at an advanced stage of HIV infection might explain the low prevalence of *H. pylori *infection in the patient group. However, the antibiotics most commonly used in AIDS patients are not always efficacious against *H. pylori*. Furthermore, low *H. pylori *eradication ratio is observed with the use of mono therapy, even with clarithromycin that has a good anti-*H. pylori *activity [29].

An interesting finding observed in this study was the presence of active chronic gastritis in the gastric body of HIV-positive patients independently of the *H. pylori *positivity, in agreement with the studies of Welage et al.; Marano et al., and Mach et al. [23,30,31], which; however, was not observed by others [6,32]. Otherwise, in this study, the *H. pylori *status was significantly associated with the presence of active chronic gastritis in the antral gastric mucosa of HIV-positive and -negative patients. Taking together the data, it is possible that different mechanisms participate in the development of corpus chronic active gastritis in HIV-positive patients. Therefore, other microorganisms such as Cytomegalovirus or some drugs used to treat AIDS and to prevent opportunistic infections may play a role [18,33].

## Conclusion

Although the prevalence of *H. pylori *infection in HIV-positive patients was lower than in HIV-negative ones, the presence of chronic active gastritis was similarly high either in HIV-positive or -negative patients, which points to the possibility that other mechanisms than *H. pylori *infection are involved in the genesis of corpus gastritis in HIV positive patients.

## Competing interests

The authors declare that they have no competing interests.

## Authors' contributions

EG: participated in the conception, performed the endoscopies, and helped writing the manuscript. MB and ABN participated in the statistical analysis, interpretation and critical writing of the manuscript. AMN: participated in implementation of the study, data collection, database management and statistical analysis. CT and CS: participated in design and implementation of the study. KC, JS and IS: participated in implementation of the study, data collection IM: statistical analysis, interpretation and writing the manuscript. DQ: performed critical writing and reviewing LB: participated in conception, design, implementation, coordination of the study and critical writing and reviewing. All authors have read and approved the final manuscript.

## Pre-publication history

The pre-publication history for this paper can be accessed here:

http://www.biomedcentral.com/1471-230X/11/13/prepub
